# Uniaxial Tensile Behavior of β-InSe Nanosheets: Thickness-Dependent Strength and Progressive Failure

**DOI:** 10.3390/nano16211353

**Published:** 2026-10-24

**Authors:** Shansong Zhou, Hongti Zhang

**Affiliations:** Shanghai Key Laboratory of High-Resolution Electron Microscopy, School of Physical Science and Technology, ShanghaiTech University, Shanghai 201210, China; zhoushs2023@shanghaitech.edu.cn

**Keywords:** InSe nanosheets, *in situ* SEM tensile testing, mechanical properties, size effect, progressive failure, nanoindentation

## Abstract

Layered indium selenide (InSe) is a promising semiconductor for optoelectronic, thermoelectric, and flexible-device applications, for which mechanical reliability is essential to device integration and operation. In this study, the mechanical behavior of β-InSe nanosheets was investigated using device-assisted *in situ* scanning electron microscopy (SEM) uniaxial tensile testing. Within the investigated thickness range, the first-instability stress generally increased with decreasing thickness, reaching a maximum measured value of 0.972 GPa, while the first-instability strain reached up to 7.383%. Notably, first instability did not lead to immediate complete fracture: some specimens continued to sustain load after initial instability, with the highest final failure strain reaching approximately 19.099%. Experimental results further showed that different portions of the nanosheet thickness fractured sequentially at different spatial locations, leading to progressive failure rather than instantaneous rupture of the entire load-bearing cross-section. These findings provide experimental insight into the thickness-dependent tensile response and progressive failure behavior of layered InSe nanosheets at the micro-/nanoscale.

## 1. Introduction

**Figure 1:**
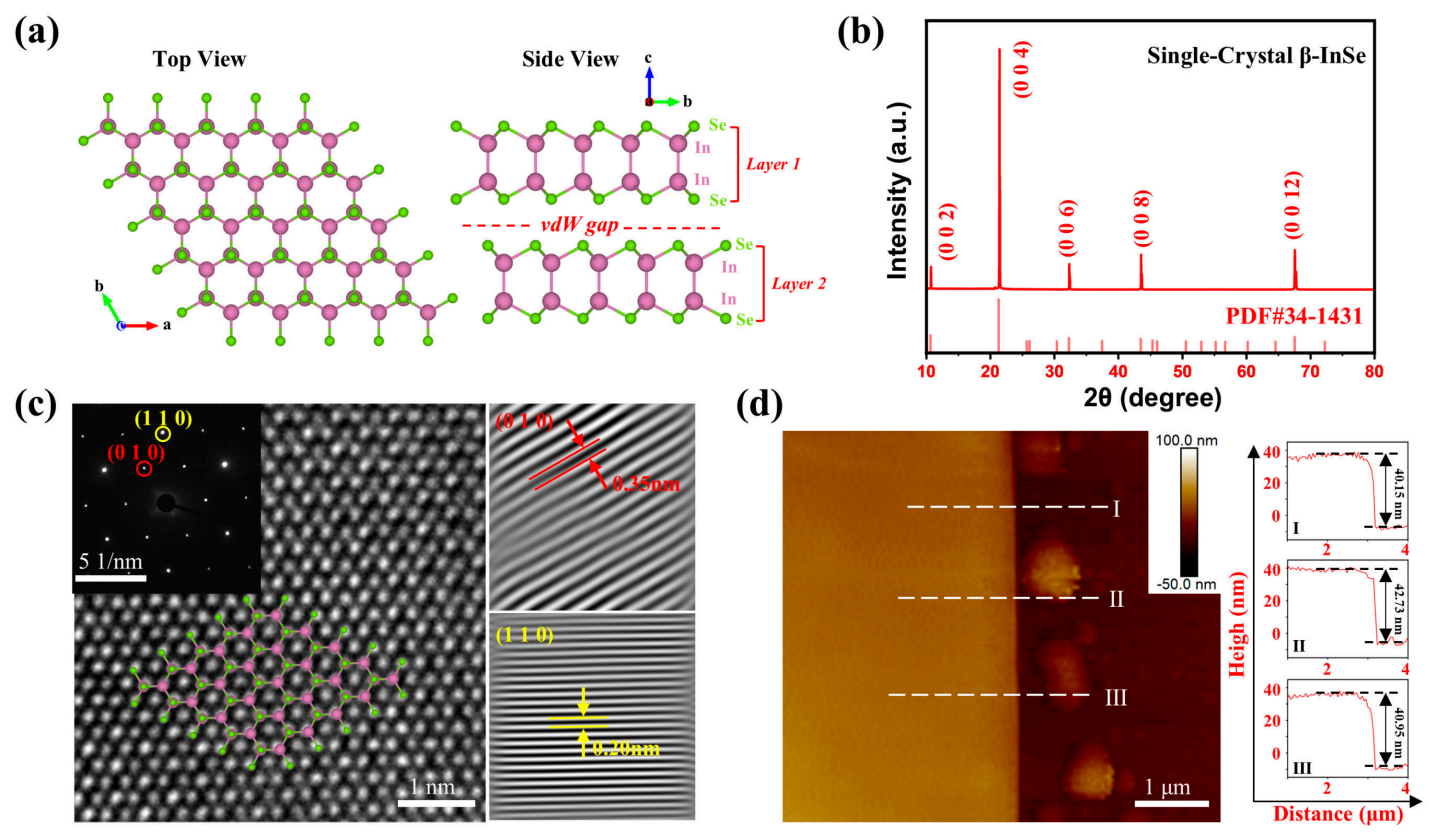
Crystal structure and material characterization of β-InSe: (**a**) schematic illustration of the β-InSe crystal structure; (**b**) XRD pattern of a β-InSe single crystal; (**c**) HRTEM image of a β-InSe single crystal, with the upper-left inset showing the corresponding SAED pattern; the IFFT images are the projections of (0 1 0) and (1 1 0) planes; (**d**) AFM thickness measurement of a β-InSe nanosheet on a PTP device, where I–III denote the three selected line-profile positions, with the corresponding height profiles shown on the right.

Two-dimensional (2D) materials have attracted extensive interest for applications in micro-/nanoelectronics [1,2], optoelectronics [3,4], thermoelectrics [5,6], and flexible devices [7,8] owing to their unique layered structures and outstanding electrical and optical properties. β-InSe is a representative group III–VI 2D layered semiconductor whose fundamental structural unit consists of four atomic planes arranged in the sequence Se-In-In-Se. Strong chemical bonding exists within each layer, whereas adjacent layers are coupled primarily through relatively weak van der Waals interactions, as illustrated in Figure 1a [9,10]. Benefiting from its distinctive layered structure and tunable electronic transport properties, InSe has been widely explored for field-effect transistors [11,12] and photodetectors [13,14] and has also emerged as a promising 2D thermoelectric material, with potential applications in energy conversion [15,16] and flexible thermoelectric devices [17,18].

However, the practical implementation of functional materials depends not only on their optoelectronic and thermoelectric performance but also on their mechanical stability. For InSe, the coexistence of strong intralayer bonding and weak interlayer interactions may give rise to interlayer sliding, delamination, and fracture under external loading. Such localized damage can further compromise structural integrity and long-term reliability during flexible deformation, device fabrication, and the temperature gradients and thermal cycling encountered in thermoelectric devices. Therefore, characterizing the mechanical properties of InSe at the micro-/nanoscale and elucidating its deformation and fracture processes are essential for understanding the relationship between its layered structure and mechanical behavior, as well as for improving the reliability of InSe-based devices.

Current studies on the mechanical properties of InSe have progressed across multiple length scales and through diverse experimental and theoretical approaches, providing increasingly detailed insights into its elasticity, strength, deformation limits, and underlying mechanisms. Li et al. investigated the mechanical properties of suspended InSe flakes by AFM indentation and obtained an in-plane Young’s modulus of ~101 GPa and a breaking strength of ~8.68 GPa [19]. In comparison, a substantially lower Young’s modulus was obtained using a buckling-based methodology [20]. Beardsley et al. further employed picosecond acoustics to probe mechanically exfoliated InSe on sapphire, revealing weak mechanical coupling across the InSe/substrate interface [21]. Theoretical calculations predicted that ideal monolayer InSe can sustain large in-plane tensile strains and undergo a strain-induced structural phase transition at high strains [22]. Beyond the elasticity and strength of thin InSe, recent studies have further revealed deformation characteristics distinct from those of conventional brittle semiconductors. Wei et al. reported exceptional room-temperature plasticity in bulk single-crystalline InSe and attributed it to the combined effects of interlayer gliding and cross-layer dislocation slip [23]. Subsequent nanoindentation, full-field strain analysis, and microstructural studies further showed that interlayer/cross-layer gliding, bond breaking and re-forming, and martensitic transformation can participate in the deformation of InSe [24,25,26]. Defects and lattice-structure changes induced by plastic deformation can also substantially modify its thermal transport behavior. Overall, these studies reveal the diverse deformation behavior of InSe and show that its mechanical response can vary considerably with material dimension and loading configuration. Although previous studies have investigated the elasticity, strength, and deformation mechanisms of InSe from different perspectives, compared with AFM nanoindentation, buckling, and micropillar compression, *in situ* uniaxial tensile testing enables direct measurement of the tensile mechanical response under a relatively well-defined in-plane stress state while allowing real-time tracking of failure evolution. Therefore, *in situ* uniaxial tensile testing of freestanding InSe nanosheets is important for further improving the understanding of their mechanical properties.

In this study, the tensile mechanical response and failure behavior of layered β-InSe nanosheets were systematically investigated using device-assisted *in situ* SEM uniaxial tensile testing combined with structural characterization. The tensile stress, strain response, and thickness dependence were quantitatively analyzed, while the force–displacement curves were correlated with the corresponding *in situ* morphological changes to examine multiple instability events and the sustained load-bearing behavior after the first instability. In addition, *in situ* nanoindentation tests were performed on bulk single-crystalline InSe to complement the characterization of its mechanical response under localized contact loading. This study aims to directly characterize the mechanical behavior of InSe nanosheets under uniaxial tension and to gain further insight into their progressive failure characteristics.

## 2. Materials and Methods

The single-crystalline β-InSe bulk specimen (PuMat™, Shanghai, China, >99% purity, approximately 3–5 mm in size) investigated in this work was purchased from PuMat™ (Shanghai, China).

### 2.1. Characterization

The crystal structure of the samples was characterized by X-ray diffraction (XRD) and transmission electron microscopy (TEM). XRD measurements were performed using a Bruker D8 Advance powder X-ray diffractometer (Bruker, Beijing, China) with Cu Kα radiation over a 2θ range of 10–80° with a step size of 0.02°. TEM characterization was conducted using a JEOL™ JEM-F200 (JEOL Ltd., Tokyo, Japan), with high-resolution transmission electron microscopy (HRTEM) and selected-area electron diffraction (SAED) employed to further analyze the lattice structure and crystallographic orientation of the samples.

### 2.2. Sample Transfer and Thickness Measurement

InSe nanosheets were first obtained from bulk single crystals by mechanical exfoliation and subsequently transferred onto Push-to-Pull (PTP) devices using a polydimethylsiloxane (PDMS)-assisted dry-transfer method. After optical microscopy screening, InSe nanosheets with suitable dimensions and morphology were selected and suspended across the gap in the working region of the PTP device. After transfer, the nanosheet thickness was measured by atomic force microscopy (AFM; Dimension FastScan, Bruker, Billerica, MA, USA) and determined from the step height between the nanosheet surface and the adjacent device surface. For each specimen, thickness measurements were acquired at three different locations, and the average value was taken as the final thickness to minimize errors arising from local surface roughness of the device and variations in measurement position. After thickness measurement, silver glue dots were applied to both ends of the nanosheet for final fixation, thereby improving the specimen-device attachment and ensuring stable load transfer during tensile testing. The assembled devices were then used for subsequent *in situ* SEM uniaxial tensile tests.

### 2.3. In Situ SEM Uniaxial Tensile Testing

The PTP device was mounted on a Hysitron Bruker™ PI 88 PicoIndenter (Bruke, Billerica, MA, USA), which was subsequently installed in the chamber of a JEOL™ JEM-IT 500 HR/LA SEM (JEOL Ltd., Tokyo, Japan). During *in situ* tensile testing, the SEM accelerating voltage was set to 10 kV, whereas a lower accelerating voltage of 3 kV was used for specimen dimension measurements prior to mechanical testing. Tensile loading was applied in displacement-control mode by moving the sample stage toward the diamond indenter. A constant displacement rate of 5 nm/s was used for all mechanical tests. During tensile loading, the diamond indenter was coupled to the force–displacement transducer, allowing the displacement and load to be recorded continuously in real time.

For quantitative analysis, the force associated with the first instability was determined from the magnitude of the corresponding abrupt load drop in the recorded load–displacement curve. The first-instability stress was calculated as

(1) $$\sigma_{\mathrm{FI}}=\frac{\Delta F_{\mathrm{First}-\mathrm{Instability}}}{w_{0}t_{0}},$$

where ΔF_First-Instability_ is the force released at the first instability, and w_0_ and t_0_ are the initial specimen width and thickness, respectively. The specimen width and gauge length were measured from low-voltage SEM images, whereas the thickness was determined by AFM as described in Section 2.2.

The specimen gauge length and global tensile elongation were determined from the *in situ* SEM images based on the change in the PTP-device gap. For specimens that were already taut before loading, the initial gauge length l_0_ was measured directly from the initial SEM image, and the global tensile elongation was calculated as

Δ*l_First_*_−*Instability*_ = *d_PTP_*_−*First*−*Instability*_ − *d_PTP_*_−*initial*_, (2)

where d_PTP-initial_ and d_PTP-First-Instability_ are the PTP-gap distances in the reference and loaded states, respectively. For specimens exhibiting initial slack, the nanosheet was first straightened before tensile deformation was evaluated. The gauge length l_0_ in the taut configuration was determined as

(3) $$l_{0}={\bar{l}}_{0}+(d_{\mathrm{PTP}-\mathrm{straightening}}-d_{\mathrm{PTP}-\mathrm{initial}}),$$

where ${\overset{-}{l}}_{0}$ is the gauge length measured in the initial configuration, and d_PTP-initial_ and d_PTP-straightening_ are the PTP-device gap distances before and after removal of the initial slack, respectively. The subsequent global tensile elongation was then calculated as

Δ*l_First_*_−*Instability*_ = *d_PTP_*_−*First*−*Instability*_ − *d_PTP_*_−*straightening*_, (4)

Accordingly, the first-instability strain was calculated as

(5) $$\varepsilon_{\mathrm{First}-\mathrm{Instability}}=\frac{\Delta l_{\mathrm{First}-\mathrm{Instability}}}{l_{0}},$$

with the displacement associated with removal of the initial slack excluded from the tensile strain. Finally, the apparent secant modulus was calculated as

(6) $$E=\frac{\sigma_{\mathrm{First}-\mathrm{Instability}}}{\varepsilon_{\mathrm{First}-\mathrm{Instability}}},$$

The uncertainties in the derived mechanical parameters were evaluated by standard first-order uncertainty propagation based on the uncertainties of the corresponding primary measurements.

### 2.4. In Situ SEM Nanoindentation Testing

High-precision nanoindentation tests were performed on bulk β-InSe inside the SEM. A cube-corner diamond indenter was used, with the indenter axis oriented normal to the (0 0 1) surface. The indentation tests were conducted under load control, with peak loads ranging from 300 to 3000 μN. Both loading and unloading times were set to 20 s, and the peak load was held for 10 s. A multi-point continuous testing mode (MPC) was employed, and measurements were performed at 10 locations on a flat region of the sample.

## 3. Results and Discussion

### 3.1. Structural and Thickness Characterization of InSe Nanosheets

As shown in Figure 1b, the XRD pattern of β-InSe is dominated by a series of (0 0 L) reflections, including the characteristic (0 0 2), (0 0 4), (0 0 6), (0 0 8), and (0 0 12) peaks. No obvious reflections from other orientations or impurity phases are observed, indicating a pronounced (0 0 L) preferred orientation, consistent with the typical layered crystal structure of β-InSe. The HRTEM image further reveals well-resolved and continuous lattice fringes (Figure 1c), while the corresponding SAED pattern exhibits sharp and regularly distributed diffraction spots, indicating a high degree of crystallographic order in the examined region. The consistent XRD, HRTEM, and SAED results confirm the good crystallinity and layered structure of the InSe samples and are consistent with the commercially specified β-InSe material, providing a reliable material basis for the subsequent micro-/nanoscale uniaxial tensile tests. Figure 1d shows the AFM topography image and corresponding height profile of a representative β-InSe nanosheet. A well-defined step is observed between the nanosheet and the adjacent device surface, yielding a thickness of approximately 41.27 nm. The thicknesses of all tensile specimens were determined using the same procedure and were used for subsequent geometric characterization and mechanical analysis.

### 3.2. Uniaxial Tensile Response of InSe Nanosheets

**Figure 2:**
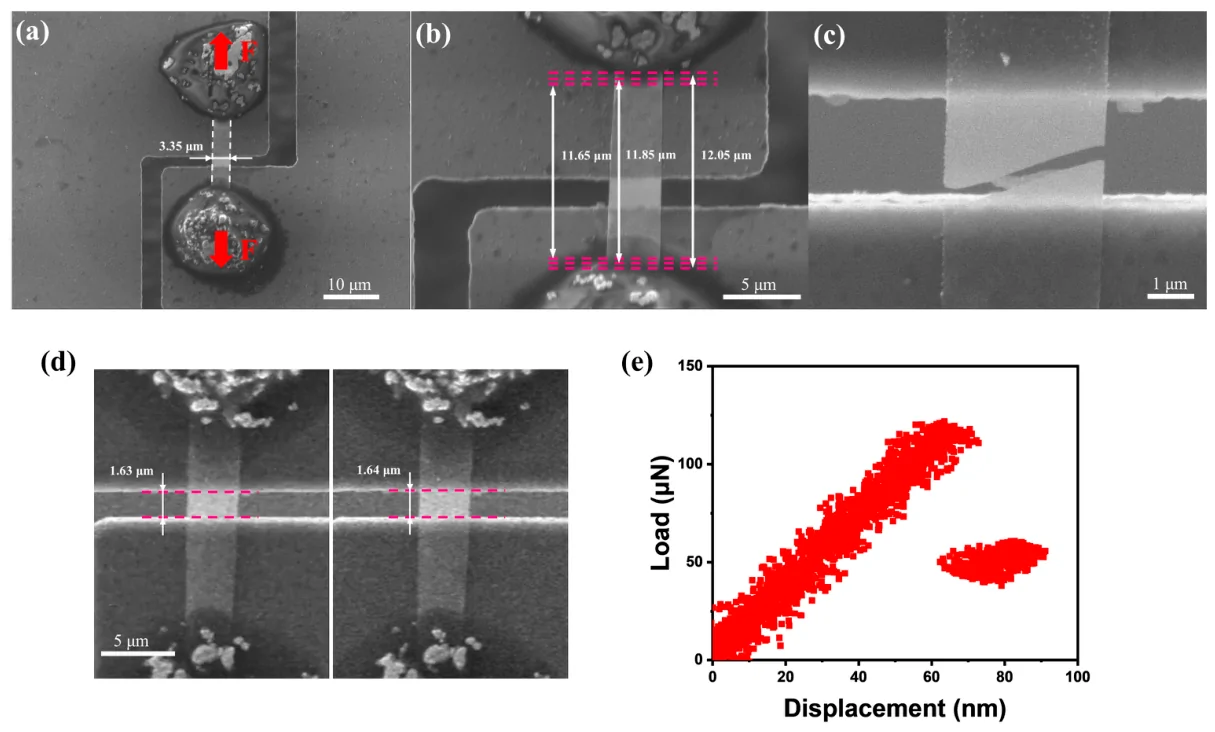
*In situ* SEM uniaxial tensile testing of β-InSe: (**a**,**b**) SEM images of a β-InSe nanosheet transferred onto a PTP device. The red arrows indicate the loading direction, and three selected positions are marked for determining the average initial gauge length of the specimen. (**c**) SEM image of the fracture morphology after fracture. (**d**) SEM frames extracted from the recorded video for determining the specimen elongation. (**e**) Corresponding load−displacement curve of the specimen.

Compared with indirect loading methods such as nanoindentation and buckling, uniaxial tensile testing enables direct characterization of the tensile mechanical response under a relatively well-defined stress state, including parameters such as apparent Young’s modulus, strength, and failure strain [27,28]. However, direct uniaxial tensile testing of micro-/nanoscale nanosheets remains experimentally challenging because of difficulties in specimen preparation and fixation, precise loading, and synchronized observation of the deformation process. In this study, InSe nanosheets were transferred onto PTP devices and subjected to *in situ* uniaxial tensile testing inside the SEM. The indenter actuated the PTP device, converting the externally applied compressive displacement into relative displacement between the two sides of the loading gap, thereby applying in-plane uniaxial tension to the suspended InSe nanosheet, as shown in Figure 2a. The experimental setup enabled simultaneous acquisition of the load–displacement response and morphological changes of the specimen, allowing characteristic features in the mechanical response to be directly correlated with the corresponding deformation and failure processes.

Figure 2 presents the geometric dimensions, morphologies before and after tensile testing, and the corresponding mechanical response of a representative InSe nanosheet. Before loading, the effective width and gauge length of the specimen were determined from SEM images (Figure 2a,b) and, together with the thickness measured by AFM, were used for subsequent stress and strain calculations. Figure 2c shows the opened fracture morphology after complete failure. Figure 2d compares the SEM images obtained before loading and immediately before final fracture, from which the actual elongation, Δ*l*, was determined based on the change in the PTP-device gap distance, and the strain was calculated using the initial gauge length, *l*_0_. For specimens exhibiting initial slack, tensile strain was evaluated once the nanosheet reached a taut configuration, with this taut configuration taken as the reference state and the displacement associated with slack removal excluded from the tensile elongation. With increasing load, the specimen first experienced a continuous loading stage, and a pronounced load drop occurred after the load reached a local maximum. For some specimens, this load drop did not immediately result in complete fracture; structural continuity was retained, and the specimen remained capable of carrying additional load. The corresponding failure process is discussed in detail below. To consistently describe the critical state associated with the first instability event among different specimens, the state corresponding to the first pronounced load drop on the load–displacement curve is defined here as the first instability, and the corresponding stress and strain are termed the first-instability stress and first-instability strain, respectively. The first instability represents the first pronounced disruption of the initially intact load-bearing state; for specimens exhibiting multiple load drops, it occurs before final complete fracture.

**Figure 3:**
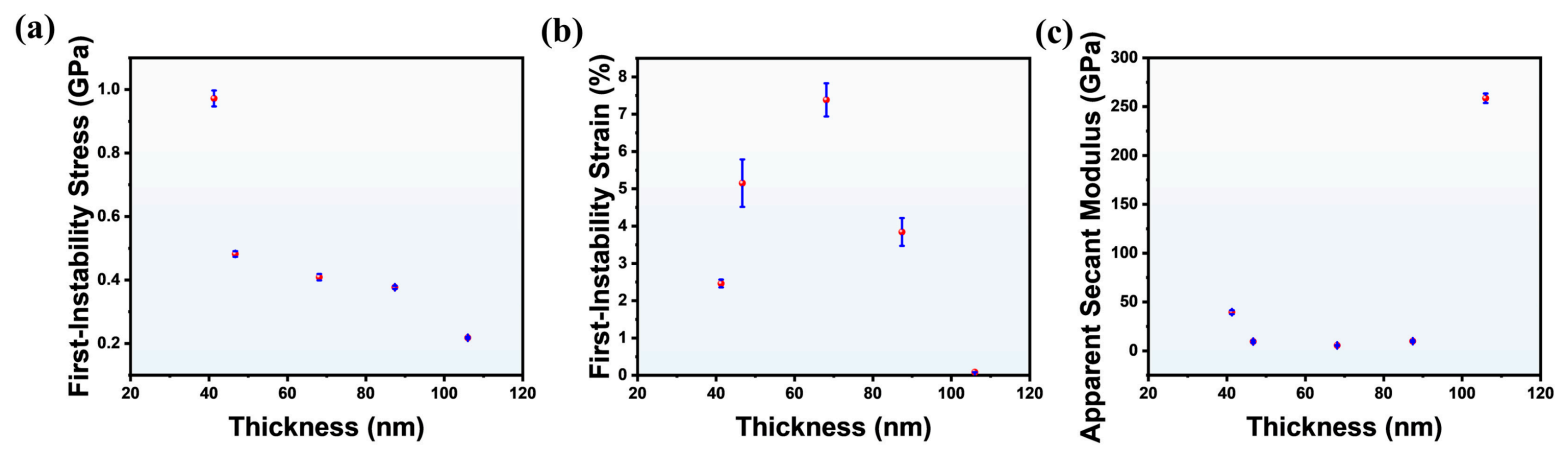
Relationship between the first-instability stress (**a**), first−instability strain (**b**), apparent secant modulus (**c**), and thickness in uniaxial tensile tests of InSe nanosheets. The blue error bars indicate the propagated measurement uncertainties.

Uniaxial tensile tests were successfully conducted on five InSe nanosheets with thicknesses ranging from approximately 41 to 106 nm. The first-instability stress ranged from 0.218 to 0.972 GPa, the first-instability strain ranged from 0.084% to 7.383%, and the corresponding apparent secant modulus ranged from 5.561 to 258.570 GPa, as shown in Figure 3 and Table 1. The corresponding geometrical parameters of the tensile specimens are summarized in Table S1 of the Supplementary Materials. Among these specimens, the nanosheet with a thickness of approximately 41.27 nm exhibited the highest first-instability stress of 0.972 GPa, whereas the specimen with a thickness of approximately 68.1 nm showed the largest first-instability strain of 7.383% and a final-instability strain of 19.10%. The mechanical responses varied considerably among the specimens, and their dependence on nanosheet thickness is discussed in the following section.

### 3.3. Thickness Dependence of the Tensile Behavior of InSe Nanosheets

Some specimens continued to carry load after the first pronounced load drop, accompanied by local delamination or partial fracture. Under these conditions, the effective load-bearing cross-sectional area after the first instability could not be determined accurately. Therefore, the first-instability stress was calculated using the intact initial cross-section before the first instability and was used to evaluate the thickness-dependent tensile strength of the InSe nanosheets. Compared with the final failure state, first instability corresponds to the critical state at which the specimen first experiences appreciable structural damage, for which the load-bearing cross-section can be determined more reliably, making it more suitable for quantitative comparison among specimens with different thicknesses. It should be noted, however, that the physical nature of the first-instability event was not identical for all specimens: in the single-drop specimens, the first instability effectively coincided with complete fracture, whereas in Samples 4 and 5, it corresponded to partial through-thickness failure. Thus, first-instability stress is used here as a consistently quantifiable comparison metric rather than as a parameter representing an identical fracture state in all specimens. As shown in Figure 3a, the first-instability stress of the InSe nanosheets generally increased with decreasing thickness over the investigated range of approximately 41–106 nm. When the thickness decreased from approximately 106 to 41 nm, the measured first-instability stress increased from 0.218 to 0.972 GPa. Although appreciable specimen-to-specimen scatter was present, the present dataset indicates an overall thickness-dependent strengthening trend, with thinner specimens generally exhibiting higher first-instability stress.

To further quantitatively describe this thickness-dependent trend, the first-instability stress was fitted using the empirical power-law relation:

σ_FI_ = K_f_ t^−n^, (7)

where σ_FI_ is the first-instability stress, t is the specimen thickness, K_f_ is the proportionality constant, and n is the fitted exponent. After logarithmic transformation of the thickness and first-instability stress, linear fitting yielded n = 1.20 ± 0.33, where the uncertainty represents the standard error of the fitted exponent, with R^2^ = 0.815. The corresponding 95% confidence interval of n was approximately 0.15–2.25. The empirical relation in the original coordinates is

σ_FI_ = 66.37 t^−1.20^, (8)

and the corresponding empirical power-law fitting curve is shown in Figure S1. The fitting indicates an overall tendency for the first-instability stress to increase with decreasing nanosheet thickness over the investigated range. However, only five valid tensile specimens were available, and appreciable scatter was present in the measured mechanical properties. Therefore, this power-law relation is used only as an empirical description of the thickness-dependent trend observed in the present dataset. Because the first-instability events correspond to different failure states in the single-drop and progressive-failure specimens, the fitted relation should not be interpreted as a statistically established, universal, or mechanism-specific scaling law for the first-instability stress.

Similar size-dependent strengthening behavior has received considerable attention in 2D materials. Previous studies have shown that factors such as thickness, defects, grain boundaries, and interlayer interactions can significantly affect the mechanical properties and fracture behavior of 2D materials [29]. For InSe specifically, atomic-resolution observations have shown that various atomic-scale defects, including metal and selenium vacancies and extended line defects, can form under electron-beam irradiation [30], further indicating the defect sensitivity of this layered material. For defect-sensitive brittle materials, reducing the specimen size may decrease the statistical probability of critical flaws being present within the effectively stressed region, thereby resulting in a higher failure strength. Accordingly, the increase in first-instability stress with decreasing thickness observed for InSe in this study is qualitatively consistent with size-dependent strengthening behavior reported for other 2D materials. Because the fracture origins and defect distributions were not directly characterized in the present study, defect-statistical considerations are regarded only as a possible explanation for the observed thickness-dependent strengthening trend. In addition, nonuniform interlayer load transfer associated with the surface-fixation configuration may also contribute to the measured thickness dependence, particularly for relatively thick specimens, and therefore cannot be completely excluded. Overall, the present results suggest that nanosheet thickness is associated with variations in the first-instability stress within the investigated range.

### 3.4. Tensile Failure Behavior of InSe Nanosheets

**Figure 4:**
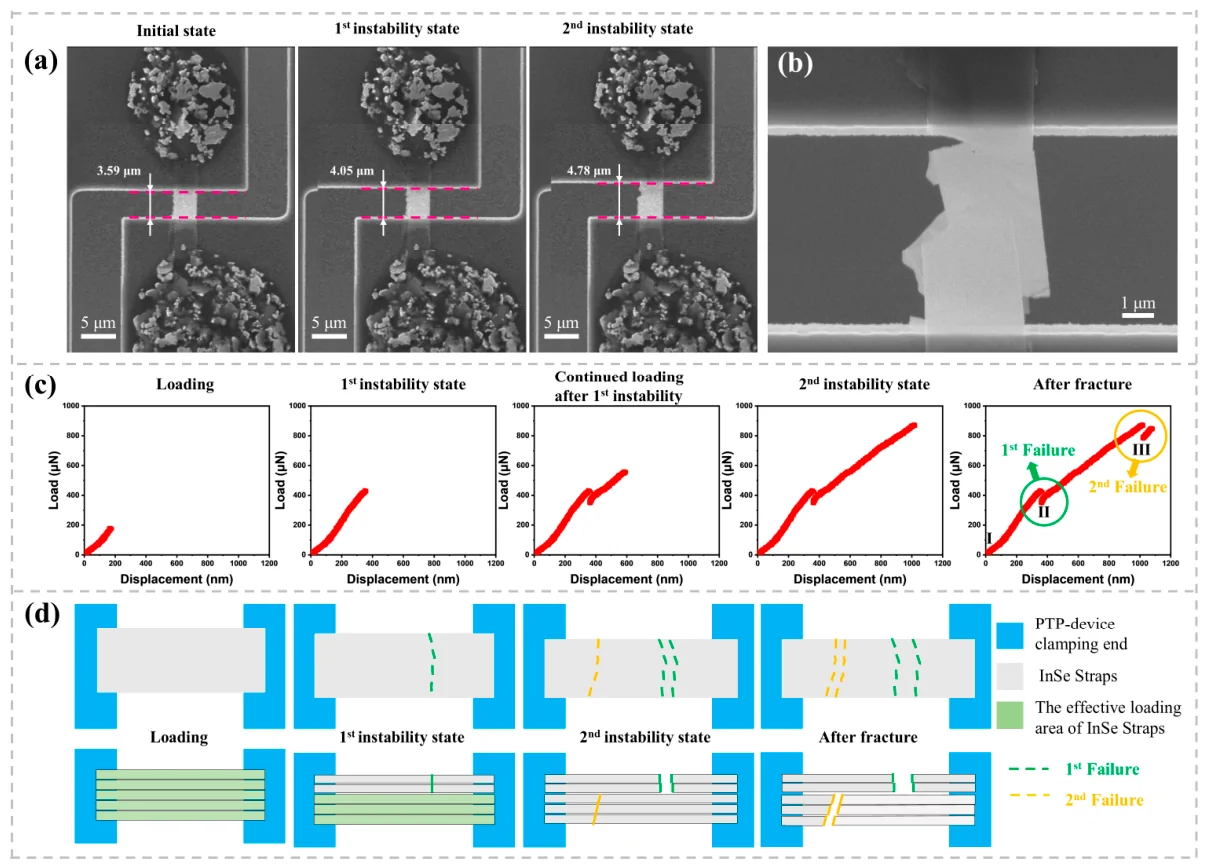
Progressive tensile failure behavior of a β-InSe nanosheet: (**a**) SEM frames extracted from the *in situ* tensile video at the initial state, immediately before first instability, and immediately before final instability, with the corresponding PTP−gap lengths marked for strain determination; (**b**) SEM image after complete fracture; (**c**) load−displacement curves with SEM frames corresponding to five characteristic stages during tensile loading; (**d**) schematic illustration of the proposed interpretation of the progressive failure process, in which different load−bearing portions across the nanosheet thickness may fail sequentially at spatially separated locations; the upper and lower schematics show the top−view and through-thickness perspectives, respectively.

*In situ* SEM results further reveal the multistage tensile failure behavior of InSe nanosheets. As shown in Figure 4, the specimen experienced two pronounced load drops. After the first load drop, the load increased again and continued to rise until the second drop, while the corresponding SEM images showed that the specimen remained structurally continuous without complete failure. Correlating the morphologies at different loading stages with the corresponding load–displacement response indicates that the first instability does not correspond to final complete fracture but rather marks the onset of the subsequent progressive failure process.

Video S1 further illustrates this multistage failure process. After the first load drop, a local fracture opening became visible, while the specimen remained structurally connected along the loading direction and continued to carry load. With further loading, additional deformation occurred, and a subsequent fracture event developed at a spatially separated location, ultimately interrupting the continuous load path. These observations indicate that the first instability does not correspond to instantaneous complete failure of the entire load-bearing cross-section. Based on the continued load carrying after the first instability, the spatial separation between successive fracture events, and the layered fracture morphology observed after failure, we propose that the failure process may involve sequential loss of different load-bearing portions through the specimen thickness. In this interpretation, part of the load-bearing thickness is disrupted during the first instability, whereas the remaining region continues to sustain load until a later instability event. The schematic in Figure 4d illustrates this possible failure scenario. Because the detailed through-thickness crack path cannot be directly resolved by the present SEM observations, this model should be regarded as a mechanistic interpretation consistent with the experimental evidence rather than as a direct reconstruction of the fracture sequence.

**Figure 5:**
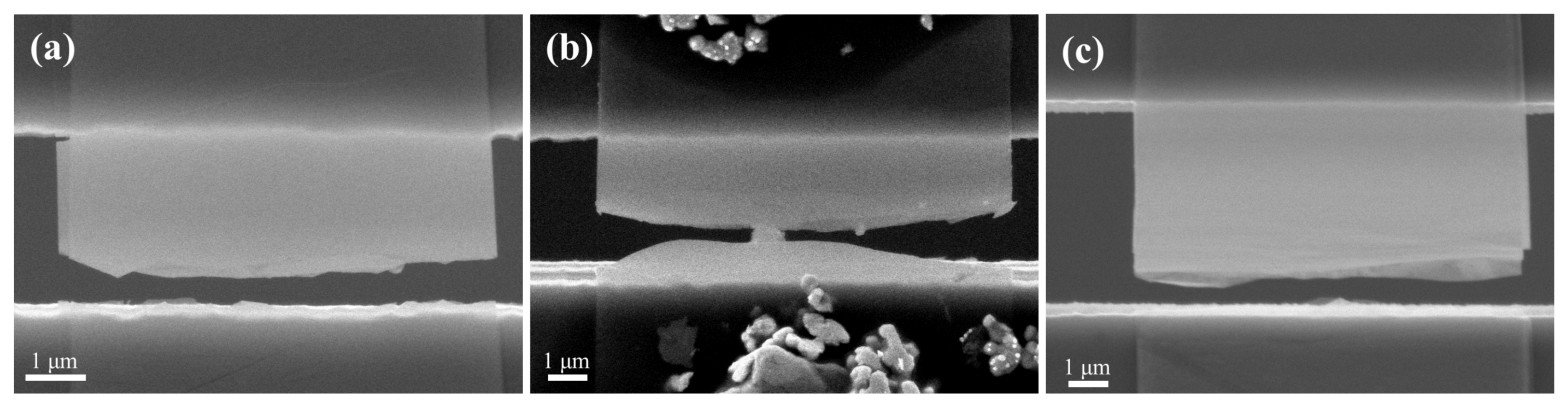
(**a**–**c**) SEM images of samples 2–4 (in Table 1) after fracture, respectively.

High-magnification SEM images after fracture revealed the fracture morphologies associated with the failure process. As shown in Figure 4b, the fracture morphology of the specimen was relatively complex, with pronounced interlayer separation and step-like fracture features. The fracture morphologies of Samples 1 and 5 are shown in Figure 2c and Figure 4b, respectively, while those of Samples 2–4 are shown in Figure 5. A comparison of the fracture morphologies of five tensile specimens after complete failure further showed that, although the fracture locations and local morphologies varied among specimens, different degrees of interlayer separation and step-like fracture features were consistently observed at the fracture regions, rather than a single flat fracture surface extending through the entire thickness. These features indicate that delamination is a relatively common morphological feature of tensile fracture in InSe nanosheets. Some local fracture-edge segments appear to exhibit angular features close to multiples of 60°; however, due to limitations in specimen-specific crystallographic orientation characterization, it is difficult to establish their direct correspondence to particular in-plane crystal directions.

The above failure characteristics may be associated with the layered structure of InSe and the difference between its intralayer and interlayer bonding. This bonding contrast may provide a structural basis for nonuniform local failure across the thickness and relative motion between adjacent layers. Within the proposed interpretation, partial loss of the load-bearing thickness may allow the remaining region to continue carrying load and undergoing further deformation, thereby contributing to the sequential fracture events observed at spatially separated locations. Meanwhile, the fracture morphologies commonly observed in the SEM images after fracture suggest that interlayer-related deformation or failure processes may contribute to the overall fracture evolution. Previous studies on multilayer 2D materials have reported that interlayer sliding, delamination, and asynchronous fracture can accompany the failure of layered structures [31,32,33,34]. The continued load-carrying capacity after the first instability, spatially separated fracture events, and layered fracture morphologies observed in the present InSe nanosheets show a broadly similar failure tendency. These similarities suggest that interlayer interactions may contribute to the overall tensile failure evolution of InSe nanosheets and influence both the delamination-related fracture morphology and the progressive failure behavior. Furthermore, such nonuniform through-thickness load transfer may also facilitate sequential failure of different portions of the nanosheet thickness and thus contribute to the observed progressive failure.

### 3.5. Nanoindentation Tests of Bulk InSe

**Figure 6:**
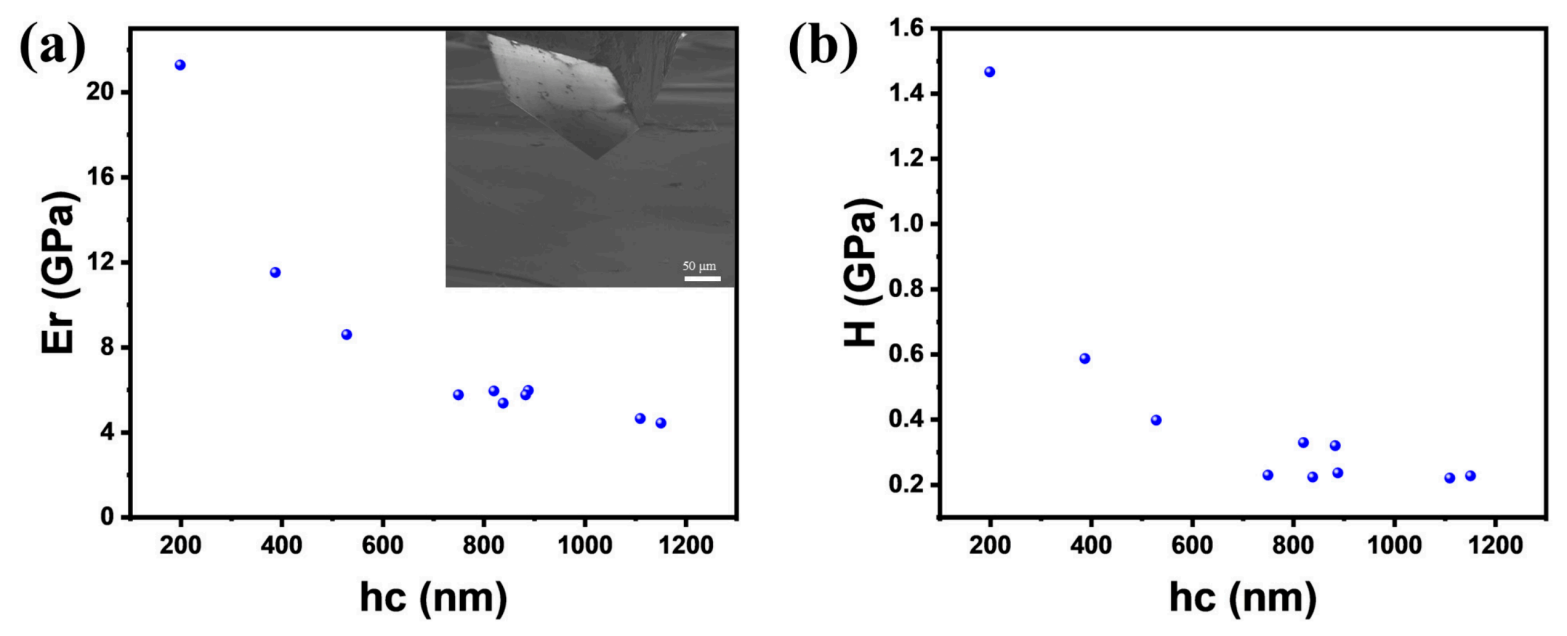
*In situ* SEM nanoindentation testing of bulk InSe: (**a**) relationship between reduced modulus E_r_ and contact depth h_c_, with an inset showing the *in situ* SEM nanoindentation experiment; (**b**) relationship between hardness H and contact depth h_c_.

To further investigate the mechanical response of InSe under localized contact loading, *in situ* SEM nanoindentation tests were performed on bulk single-crystalline InSe, as shown in Figure 6. The hardness and reduced modulus generally decreased with increasing contact depth and gradually approached relatively stable values, indicating a pronounced indentation-depth dependence. This behavior may be associated with the increasing material volume involved in deformation and the local response of the layered structure with increasing indentation depth. Unlike in-plane uniaxial tension, nanoindentation imposes a localized multiaxial stress state, so the resulting mechanical parameters are not directly comparable. Together, the two tests highlight the dependence of the mechanical response of InSe on loading configuration and length scale.

### 3.6. Comparison with Previous Mechanical Studies of InSe

Table 2 summarizes representative experimental and simulation studies on the mechanical properties of InSe reported in recent years. Previous studies have mainly focused on AFM nanoindentation, buckling, bulk compression, bending, and atomistic simulations, revealing mechanical characteristics of InSe such as low stiffness, high flexibility, pronounced plasticity, and interlayer sliding. However, systematic experimental investigations of suspended micro-/nanoscale InSe nanosheets under direct in-plane uniaxial tension are still relatively scarce, particularly regarding the thickness dependence of the tensile response and the corresponding failure evolution under this loading configuration. The present study complements these previous efforts by providing direct tensile measurements over the investigated thickness range and by identifying thickness-dependent first-instability behavior together with progressive failure accompanied by delamination features.

The mechanical parameters reported for InSe also vary considerably among different studies, further reflecting the influence of specimen characteristics and testing configurations on its measured mechanical response. For example, AFM nanoindentation measurements yielded an in-plane apparent Young’s modulus of approximately 101 GPa, whereas a value of approximately 23 GPa was obtained using a buckling-based method. By comparison, the apparent secant modulus measured by direct uniaxial tensile testing in the present study ranged from 5.561 to 258.570 GPa. The measured values are influenced by factors such as specimen thickness and structural state, loading configuration, and boundary conditions. Therefore, mechanical parameters obtained from different studies should not be compared solely on a numerical basis without considering the corresponding experimental conditions. In this context, the present study complements these previous efforts by showing an overall thickness-dependent trend in first-instability stress together with progressive failure accompanied by delamination features.

## 4. Conclusions

In summary, we found that β-InSe nanosheets exhibit pronounced thickness-dependent tensile behavior and progressive failure under direct in-plane uniaxial loading. Over the investigated thickness range of approximately 41–106 nm, the first-instability stress ranged from 0.218 to 0.972 GPa, the first-instability strain from 0.084% to 7.383%, and the apparent secant modulus from 5.561 to 258.570 GPa. Within this thickness range, the first-instability stress generally increased with decreasing thickness, indicating an overall thickness-dependent strengthening trend. For some specimens, first instability did not lead to immediate complete fracture; instead, the remaining load-bearing regions continued to carry load and undergo further deformation. Subsequent fracture events occurred at spatially separated locations, demonstrating a progressive rather than instantaneous failure process. Together with the observed interlayer separation and step-like fracture morphologies, these features are consistent with a possible sequential failure of different load-bearing portions through the nanosheet thickness. Nanoindentation of bulk InSe further showed pronounced indentation-depth dependence of both hardness and reduced modulus. These findings contribute to the understanding of the mechanical behavior of layered InSe under different loading configurations and provide a useful basis for the structural design and mechanical reliability assessment of InSe-based micro-/nanodevices.

Orientation-dependent mechanical anisotropy may also contribute to specimen-to-specimen variation in the measured tensile response. The role of crystallographic orientation in the tensile response and failure mechanisms of InSe nanosheets could be elucidated in future orientation-resolved mechanical investigations.

## Figures and Tables

**Table 1:** Summary of uniaxial tensile mechanical properties of InSe nanosheets.

Sample	Thickness/nm	First-Instability Stress (GPa)	First-Instability Strain (%)	Final-Instability Strain (%)	Apparent Secant Modulus (GPa)	Strain Rate (s^−1^)
1	105.99	0.218 ± 0.003	0.084 ± 0.001	/	258.570 ± 4.833	5.88 × 10^−5^
2	41.27	0.972 ± 0.025	2.462 ± 0.105	/	39.558 ± 1.993	3.96 × 10^−4^
3	46.71	0.482 ± 0.009	5.151 ± 0.637	/	9.492 ± 1.167	1.78 × 10^−3^
4	87.40	0.377 ± 0.005	3.842 ± 0.372	5.361 ± 0.520	9.909 ± 0.960	4.56 × 10^−4^
5	68.12	0.409 ± 0.010	7.383 ± 0.445	19.099 ± 1.152	5.561 ± 0.361	1.02 × 10^−3^

Note: “/” indicates that no separate final-instability event was identified after the first instability. The apparent secant modulus of Sample 1 has relatively large uncertainty because it is derived from a very small measured elongation (~10 nm) close to the displacement-measurement resolution.

**Table 2:** Representative studies on the mechanical behavior of InSe.

Ref.	Sample Configuration	Dimension	Method	Loading mode	Main Mechanical Properties	Key Finding
Hu et al. [22]	Monolayer InSe	Monolayer	First-principles calculations	In-plane strain	Tensile strain up to 27% along the armchair direction.	Large intrinsic strain tolerance and strain-induced structural reconstruction.
Li et al. [19]	Suspended multilayer InSe flakes	>5 layers	AFM Nanoindentation, FEM and DFT	Out-of-plane indentation	E = 101.37 ± 17.93 GPa; breaking strength = 8.68 GPa.	Quantified the elastic modulus and breaking strength of suspended multilayer InSe.
Zhao et al. [20]	Thin InSe flakes supported on PDMS	~1–2 to ~34 layers	Buckling-based metrology	Compression-induced buckling	E = 23.1 ± 5.2 GPa.	Demonstrated the relatively low stiffness and high flexibility of thin InSe.
Wei et al. [23]	Bulk β-InSe and micropillars	Bulk/microscale	Micropillar compression and roller pressing tests	Compression along and perpendicular to the c-axis	~80% compressive strain along and perpendicular to the c-axis; >4000% thickness reduction by roller pressing; bendable into a Möbius strip or origami without fracture.	InSe plasticity: interlayer gliding + cross-layer slip, mediated by interlayer Coulomb interaction & soft bonding.
Ma et al. [24]	Polymorphic bulk InSe vdW crystals	Bulk	Macroscopic mechanical tests, nanoindentation and DFT calculations	Tension/compression/bending/indentation	Pronounced plastic deformation; low modulus and hardness.	All InSe polymorphs show plasticity/softness via interlayer & cross-layer slip with bond breaking/reforming.
Ma et al. [25]	Bulk InSe van der Waals crystals	Bulk	In-situ DIC compression	Compression along the c-axis	Yielding at ~0.7% strain; compressible to 50% strain; local strain up to ~12% at ε_e_ = 4.4%.	Plastic deformation via interlayer and cross-layer glides, directly evidenced by DIC.
Sun et al. [26]	β-InSe pillars	Nano/micropillars (simulation/experiment)	MD simulations (DP) and compression	Out-of-plane compression	Residual strain ~12% (simulation); ~50% strain (experiment)	Identified martensitic transformation as an additional plastic-deformation mechanism.
This Work	Single-crystal InSe nanosheets	~41–106 nm	*In situ* tensile and nanoindentation	In-plane uniaxial tension/out-of-plane indentation	First-instability stress: 0.218–0.972 GPa; first-instability strain: 0.084–7.383%; apparent secant modulus: 5.561–258.570 GPa.	Thickness-dependent strength and asynchronous progressive failure.

## Data Availability

The original contributions presented in the study are included in the article/Supplementary Material. Further inquiries can be directed to the corresponding author.

## Supplementary Materials

The following supporting information can be downloaded at https://www.mdpi.com/article/10.3390/nano16211353/s1, Figure S1. Thickness dependence of the first-instability stress shown on logarithmic axes, together with the empirical power-law fit σ_FI_ = 66.37 t^−1.20^; Table S1. Geometrical parameters of the tensile specimens in Table 1; Video S1: InSe nanosheets *in situ* uniaxial tensile test.

## References

[1] Wang Y., Sarkar S., Yan H., Chhowalla M. (2024). Critical challenges in the development of electronics based on two-dimensional transition metal dichalcogenides. Nat. Electron..

[2] Liu C., Jiang Y., Shen B., Yuan S., Cao Z., Bi Z., Wang C., Xiang Y., Wang T., Wu H. (2025). A full-featured 2D flash chip enabled by system integration. Nature.

[3] Ren K., Pan Z., Tu X., Pan H., Gao K., Li Z., Chu H., Fang Y., Li D. (2025). 2D Transition Metal Carbo-Chalcogenide Nb2CSe2: A Combination of Transition Metal Dichalcogenide and MXene for Ultrafast Photonics Application. Adv. Opt. Mater..

[4] Han J., Fu Z., Wei J., Han S., Deng W., Hu F., Wang Z., Zhou H., Yu H., Gou J. (2025). 2D materials-based next-generation multidimensional photodetectors. Light Sci. Appl..

[5] Deng T., Gao Z., Qiu P., Wei T.-R., Xiao J., Wang G., Chen L., Shi X. (2022). Plastic/Ductile Bulk 2D van der Waals Single-Crystalline SnSe2 for Flexible Thermoelectrics. Adv.Sci..

[6] Liu Z., Deng T., Qiu P., Zheng W., Li Z., Zhou Z., Shi X. (2024). Mechanical and Thermoelectric Properties of Na-Intercalated SnSe2 Crystals. ACS Appl. Energy Mater..

[7] Huang X., Wu L., Hu M., Feng X., Zhai P., Li W., Duan B., He J., Li G., Zhang Q. (2025). The staggered-layer induced elasticity strengthening mechanism in flexible Bi2Te3. Matter.

[8] Peng Y., Cui C., Li L., Wang Y., Wang Q., Tian J., Huang Z., Huang B., Zhang Y., Li X. (2024). Medium-scale flexible integrated circuits based on 2D semiconductors. Nat. Commun..

[9] Popovic S., Tonejc A., Grzeta-Plenkovic B., Celustka B., Trojko R. (1979). Revised and new crystal data for indium selenides. J. Appl. Crystallogr..

[10] Wu J., Lin Y., Shu M., Liu Y., Ma Y., Lin G., Zhang C., Jiao P., Zhu F., Wu Y. (2024). Uncovering the phonon spectra and lattice dynamics of plastically deformable InSe van der Waals crystals. Nat. Commun..

[11] Jiang J., Xu L., Qiu C., Peng L.-M. (2023). Ballistic two-dimensional InSe transistors. Nature.

[12] Qin B., Jiang J., Wang L., Guo Q., Zhang C., Xu L., Ni X., Yin P., Peng L.-M., Wang E. (2025). Two-dimensional indium selenide wafers for integrated electronics. Science.

[13] Li L., Wu Q., Wang C., Cai Z., Lin L., Gu X., Ostrikov K.K., Nan H., Xiao S. (2024). High-Performance InSe Photodetector Induced by Synergetic Surface Plasmon Resonance and Surface Engineering. ACS Photonics.

[14] Zhao D., Chen Y., Hu T., Cao H., Zhao X., Jia Y., Wang X., Shen H., Yang J., Zhang Y. (2025). Bilateral Geiger mode avalanche in InSe Schottky photodiodes. Nat. Commun..

[15] Shi H., Wang D., Xiao Y., Zhao L.-D. (2021). Dynamic carrier transports and low thermal conductivity in n-type layered InSe thermoelectrics. Aggregate.

[16] Shi H.-N., Bai S.-L., Wang Y.-P., Su L.-Z., Cao Q., Chang C., Zhao L.-D. (2024). Contrasting strategies of optimizing carrier concentration in bulk InSe for enhanced thermoelectric performance. Rare Met..

[17] Liu Q.-Y., Shi X.-L., Cao T.-Y., Chen W.-Y., Li L., Chen Z.-G. (2025). Advances and challenges in inorganic bulk-based flexible thermoelectric devices. Prog. Mater. Sci..

[18] Shankar M.R., Prabhu A.N., Nayak R. (2025). Enhancing the Power Output of InSe-Based Screen-Printed Flexible Thermoelectric Generators through a Bi–Te–Co-Doping Strategy. ACS Omega.

[19] Li Y., Yu C., Gan Y., Kong Y., Jiang P., Zou D.F., Li P., Yu X.F., Wu R., Zhao H. (2019). Elastic properties and intrinsic strength of two-dimensional InSe flakes. Nanotechnology.

[20] Zhao Q., Frisenda R., Wang T., Castellanos-Gomez A. (2019). InSe: A two-dimensional semiconductor with superior flexibility. Nanoscale.

[21] Beardsley R., Akimov A.V., Greener J.D.G., Mudd G.W., Sandeep S., Kudrynskyi Z.R., Kovalyuk Z.D., Patanè A., Kent A.J. (2016). Nanomechanical probing of the layer/substrate interface of an exfoliated InSe sheet on sapphire. Sci. Rep..

[22] Hu T., Zhou J., Dong J. (2017). Strain induced new phase and indirect–direct band gap transition of monolayer InSe. Phys. Chem. Chem. Phys..

[23] Wei T.-R., Jin M., Wang Y., Chen H., Gao Z., Zhao K., Qiu P., Shan Z., Jiang J., Li R. (2020). Exceptional Plasticity in the Bulk Single-Crystalline van Der Waals Semiconductor InSe. Science.

[24] Ma Y., Huang H., Liu Y., Chen H., Bai X., Zhao K., Jin M., Wei T.-R., Shi X. (2023). Remarkable plasticity and softness of polymorphic InSe van der Waals crystals. J. Mater..

[25] Ma Y., Pan Z., Liu Y., Zhao K., Xiao J., Nam D., Wei T.-R., Shi X. (2024). Spatio-temporal strain analysis and thermal transport modulation in plastically deformed InSe van der Waals crystals. Mater. Today Energy.

[26] Sun Y., Ma Y., Zhang J.-Y., Wei T.-R., Shi X., Rodney D., Xu B. (2024). Van Der Waals Semiconductor InSe Plastifies by Martensitic Transformation. Sci. Adv..

[27] Zhang H., Tersoff J., Xu S., Chen H., Zhang Q., Zhang K., Yang Y., Lee C.-S., Tu K.-N., Li J. (2016). Approaching the ideal elastic strain limit in silicon nanowires. Science.

[28] Bai T., Wang B., Xing Y., Huang H., Chen H., Zhang H. (2026). Intrinsic and External Factors Influencing the Ultralarge Elasticity of Silicon Nanowires. Small.

[29] Jiang H., Zheng L., Liu Z., Wang X. (2020). Two-dimensional materials: From mechanical properties to flexible mechanical sensors. InfoMat.

[30] Hopkinson D.G., Zólyomi V., Rooney A.P., Clark N., Terry D.J., Hamer M., Lewis D.J., Allen C.S., Kirkland A.I., Andreev Y. (2019). Formation and Healing of Defects in Atomically Thin GaSe and InSe. ACS Nano.

[31] Zhang X., Zhao C., Wang B., Zhang D., Bai T., Wu Y., Cao K., Li J., Zhang H. (2025). Reduced Fracture Anisotropy yet Enhanced Deformability of Multilayer MoS2 Straps. ACS Appl. Mater. Interfaces.

[32] Li B., Li J., Jiang W., Wang Y., Wang D., Song L., Zhu Y., Wu H., Wang G., Zhang Z. (2024). Anisotropic Fracture of Two-Dimensional Ta2NiSe5. Nano Lett..

[33] Li P., Cao K., Jiang C., Xu S., Gao L., Xiao X., Lu Y. (2019). In situ tensile fracturing of multilayer graphene nanosheets for their in-plane mechanical properties. Nanotechnology.

[34] Jang B., Kim B., Kim J.-H., Lee H.-J., Sumigawa T., Kitamura T. (2017). Asynchronous cracking with dissimilar paths in multilayer graphene. Nanoscale.

