# Supplementary material for: Uniaxial Tensile Behavior of β-InSe Nanosheets: Thickness-Dependent Strength and Progressive Failure
Source: Nanomaterials (Basel). 2026 Oct 24;16(21):1353. doi: 10.3390/nano16211353 (PMC13764821; doi:10.3390/nano16211353)
Supplement: Supplementary file 1 [file nanomaterials-16-01353-s001.zip › Supplementary Materials.pdf]

nanomaterials

Supplementary Materials

Uniaxial Tensile Behavior of β-InSe Nanosheets: Thickness-Dependent Strength and Progressive Failure

Shansong Zhou and Hongti Zhang *

Shanghai Key Laboratory of High-Resolution Electron Microscopy, School of Physical Science and Technology, ShanghaiTech University, Shanghai 201210, China; zhoushs2023@shanghaitech.edu.cn
* Correspondence: zhanght3@shanghaitech.edu.cn

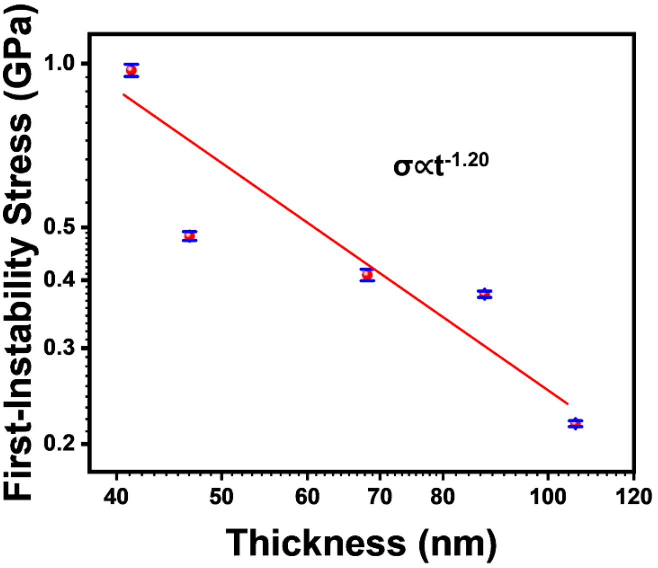

Figure S1. Thickness dependence of the first-instability stress shown on logarithmic axes, together with the empirical power-law fit $\sigma_{FI} = 66.37\ t^{-1.20}$.

Table S1. Geometrical parameters of the tensile specimens in Table 1.

Sample	Width/μm	Length/μm	$d_{PTP\text{-}initial}$/μm	$d_{PTP\text{-}straightening}$/μm	$d_{PTP\text{-}First\text{-}Instability}$/μm	$d_{PTP\text{-}Final\text{-}Instability}$/μm
1	3.35	11.85	1.63	/	1.64	/
2	7.29	11.39	1.66	1.66	1.94	/
3	9.98	2.88	1.51	1.59	1.74	/
4	9.59	10.92	3.43	3.81	4.24	4.41
5	3.13	6.25	3.59	/	4.05	4.78

"/" in $d_{PTP\text{-}straightening}$ indicates that no straightening was required; "/" in $d_{PTP\text{-}Final\text{-}Instability}$ indicates that no separate final-instability event occurred after the first instability.
